# Effects of glycemic control and hypoglycemia on Thrombus formation assessed using automated microchip flow chamber system: an exploratory observational study

**DOI:** 10.1186/s12959-019-0206-8

**Published:** 2019-09-02

**Authors:** Kiyoaki Yamamoto, Takashi Ito, Tomoka Nagasato, Atsushi Shinnakasu, Mihoko Kurano, Aiko Arimura, Hiroshi Arimura, Hiroshi Hashiguchi, Takahisa Deguchi, Ikuro Maruyama, Yoshihiko Nishio

**Affiliations:** 1 Department of Diabetes and Endocrine Medicine, Kagoshima University Graduate School of Medical and Dental Sciences, 8-35-1, Sakuragaoka, Kagoshima, 890-8544 Japan; 2 Department of Systems Biology in Thromboregulation, Kagoshima University Graduate School of Medical and Dental Sciences, 8-35-1, Sakuragaoka, Kagoshima, 890-8544 Japan; 3 Fujimori Kogyo Co., Research Institute, 1-10-1, Yokohama, Kanagawa, 236-0003 Japan

**Keywords:** Glycemic control, Hyperglycemia, Hypoglycemia

## Abstract

**Background:**

Thrombus formation is an important factor affecting cardiovascular events and venous thromboembolism in type 2 diabetes. However, it is unclear whether glycemic control reduces thrombogenicity. We investigated the effect of short-term glycemic control (STUDY 1) and hypoglycemia (STUDY 2) on thrombus formation using an automated microchip flow chamber system.

**Methods:**

For STUDY 1, we recruited 10 patients with type 2 diabetes. Before and after 2 weeks of treatment, blood glucose was analyzed with a continuous glucose monitoring system, and thrombogenicity was analyzed with an automated microchip flow chamber system. For STUDY 2, we recruited 10 subjects without diabetes who underwent an insulin tolerance test. We evaluated the change in thrombogenic potential with hypoglycemia.

**Results:**

STUDY1: The mean blood glucose level reduced from 10.1 ± 2.6 to 6.9 ± 0.97 mM (*P* < 0.01). T10, an indicator of thrombogenicity, significantly attenuated after glycemic control (338 ± 65 vs. 425 ± 117 s, *P* < 0.05). The attenuation in T10 was significantly correlated with changes in mean blood glucose level after treatment (*r* = − 0.718, *P* < 0.05). STUDY 2: Platelet function was enhanced with decreasing blood glucose; increased platelet function was strongly correlated with an increase in epinephrine.

**Conclusions:**

We demonstrated attenuation in thrombogenicity with short-term comprehensive diabetes care and enhancement in thrombogenicity with hypoglycemia, using a new flow chamber system.

**Trial registration:**

UMIN-CTR UMIN 000019899, registered 26-Jan-2015 (STUDY 2).

**Electronic supplementary material:**

The online version of this article (10.1186/s12959-019-0206-8) contains supplementary material, which is available to authorized users.

## Background

Atherothrombotic disease is a major cause of death in patients with diabetes [1]. The mechanism of increased thrombosis in patients with diabetes involves multiple pathways. Thrombus formation, the last step in the atherothrombotic process, is an important factor influencing the risk and prognosis associated with cardiovascular events [2]. Moreover, diabetes is a risk factor for arterial and venous thrombosis. A recent meta-analysis found that patients with diabetes were at increased risk of developing venous thromboembolism (VTE), which can occur in a hypercoagulable state [3].

Several studies have shown that patients with diabetes have increased thrombogenicity owing to platelet hyperreactivity, activation of coagulation factors and hypo-fibrinolysis [4–6]. However, it is unclear whether glycemic control reduces thrombus formation. Especially, the influence of glycemic control over short periods of time, such as one or two weeks, has not been assessed, even though it is a clinically-important issue for preventing post-surgical complications [7].

On the other hand, evidence is accumulating that hyperglycemia and hypoglycemia, which can occur when trying to achieve strict glycemic control, is associated with increased risk of cardiovascular and cerebrovascular death. In the ACCORD study, the mortality rate was higher in the strict-treatment than in the standard-treatment group [8]. The involvement of hypoglycemia as a risk factor for thrombosis is a topic of active discussion.

A previous study evaluated thrombogenicity using plasma markers [9] and platelet function tests with an agonist, above physiological concentrations; however, the effects of blood flow were not considered and blood flow is a well-known influence on the thrombus formation process [10]. Therefore, it is necessary to evaluate thrombogenicity comprehensively, using close-to-physiological conditions and considering blood flow. Recently, a Total Thrombus formation Analysis System (T-TAS, Fujimori Kogyo Co., Yokohama, Kanagawa) [11] was found useful [12] for quantitative analysis of thrombus formation. The T-TAS continuously monitors the pressure on microchips with thrombogenic surfaces that simulate pathological blood vessels [11, 13]. Compared with conventional systems, the T-TAS can evaluate thrombogenicity under blood flow conditions; therefore, it is possible to perform analyses that more closely resemble in vivo conditions.

This pilot study aimed at investigating the influence of short-term glycemic control (STUDY 1) and change in hypoglycemia (STUDY 2) on thrombus formation using T-TAS.

## Materials and methods

### Study 1

Glycemic control efforts were started immediately after hospitalisation. Blood glucose levels were controlled with insulin, GLP-1 analogues and oral hypoglycemic agents. The goal of treatment was to achieve a fasting plasma glucose (FPG) level < 7.2 mM and a postprandial PG level < 11.1 mM. A continuous glucose monitoring (CGM) device (iPro2: Medtronic Minimed, Northridge, CA, USA) was attached to each patient from the day of hospitalization, and monitoring was performed for 3 days. Blood collection and evaluation for T-TAS were carried out on the day after hospitalization. These evaluations were performed again after glycemic control was achieved. The exclusion criteria were antiplatelet drug or anticoagulant use, infectious diseases, malignant tumors, drug-induced diabetes, ketoacidosis and possible pregnancy.

### Blood samples

Following overnight fasting, blood was drawn for the measurement of blood glucose, hematocrit (Ht), platelet, plasminogen activator inhibitor 1 (PAI-1), fibrinogen, factor VII (FVII), factor VIII (FVIII) and prothrombin fragment 1 + 2 (F1 + 2), and for assessment using T-TAS.

Glucose levels were measured using a GA echo buffer solution, Glucose BP standard solution and automated glucose analyzer GA08III (A&T corporation, Kanagawa, Japan). The LPIA·tPAI test with the automated immunoanalyzer STACIA (LSI Medience Corporation, Tokyo, Japan) for PAI-1, Thrombo check Fib (L) with CS-5100 Automated Coagulation Analyzer (Sysmex Corporation, Hyogo, Japan) for fibrinogen, HemosIL RecombiPlasTin or HemosIL Synth Asil with ACL TOP automated analyzer (Instrumentation Laboratory, Bedford, MA) for FVII or FVII and Enzygnost with Microplate Reader Emax (Molecular Devices Japan, Tokyo, Japan) for F1 + 2 were applied respectively.

### CGM

We used a CGM device and considered the result as an indicator of short-term glycemic control. The mean blood glucose level, peak blood glucose levels and standard deviation (SD) and mean amplitude of glycemic excursion (MAGE) were calculated from the results.

### T-TAS

T-TAS evaluations were carried out using two kinds of microchips (PL chip and AR chip). The PL chip, which is coated with collagen, represents platelet thrombus formation. The AR chip, which is coated with collagen plus tissue thromboplastin, represents thrombus formation mediated by the coagulo-fibrinolysis system and platelets. Whole blood anticoagulated with hirudin was perfused through the PL chip at a shear rate of 1500 S^− 1^; then, PL-T10 (time to 10 kPa), PL-OT (occlusion time: time to 60 kPa) and PL-AUC10 (area under the flow curve in 10 min) were used to evaluate thrombus formation. Recalcified whole blood containing corn trypsin inhibitor was perfused through the AR chip at a sheer rate of 600 S^− 1^; then, AR-T10 (time to 10 kPa), AR-OT (occlusion time: time to 80 kPa) and AR-AUC30 (area under the flow curve in 30 min) were evaluated. The intra-assay coefficient of variability of PL-AUC and AR-AUC are 5.85 and 1.24% respectively (Additional file 1: Figure S1). The reference ranges (mean ± SD) of PL-T10, PL-OT and PL-AUC in healthy volunteers were reported to be 163 ± 51 s, 347 ± 96 s, and 369.1 ± 71.8 kPa × min, respectively [14].

### Study 2

Human regular insulin (Eli Lilly, Indianapolis, IN) was injected intravenously at a dose of 0.05–0.10 U/kg body weight. Blood samples for glucose and epinephrine were collected before the injection and at 15, 30, 45, 60, 90 and 120 min after the injection; for T-TAS blood samples were collected before the injection and at 15, 30, 60, 90 and 120 min; and for WBC blood samples were collected before the injection and at 30 and 60 min after the injection. For measurement of epinephrine, the CA Test 「TOSOH」 (Tosoh Corporation, Tokyo, Japan) and HPLC system (SHIMADZU CORPORATION, Kyoto, Japan) were used.

#### Statistical analysis

Normally distributed data are presented as means ± SDs, and the paired *t*-test was used to assess the presence of significant differences. Non-normally distributed data are presented as medians (quartile range), and the Wilcoxon signed rank test was used for assessment. Spearman’s rank correlation was used for assessing correlation. IBM SPSS 20 statistical package (IBM Corp., Armonk, NY) was used to perform all the statistical analyses. A *P*-value < 0.05 was considered statistically significant.

## Results

### Study 1

We included 10 patients with type 2 diabetes who were admitted to Kagoshima University Hospital for glycemic control (Table 1) (Additional file 1: Table S1). The mean duration of the period between pre- and post-glycemic control was 10.4 days. The levels of fasting plasma glucose (FPG) reduced from 9.6 ± 2.3 mM to 6.3 ± 1.4 mM (*P* < 0.01), mean blood glucose reduced from 10.1 ± 2.5 mM to 6.8 ± 0.9 mM (*P* < 0.01), and peak blood glucose reduced from 15.6 ± 4.3 mM to 11.5 ± 4.1 mM (*P* < 0.05). The SD of blood glucose and MAGE were unchanged (2.1 ± 1.3 mM vs. 1.6 ± 1.2 mM, *P* = 0.24 and 87.5 ± 42.4 vs. 68.5 ± 51.2, *P* = 0.31, respectively) (Table 2). In one case, mild hypoglycemia (3.1 mM) was recognized in the night during CGM; however, this was not observed in other patients.

**Table 1:** Patient characteristics in STUDY 1

Age (years)	47.5 ± 16.6
Sex (male/female)	5 / 5
BMI (kg/m^2^)	29.6 ± 7.9
FPG (mM)	10.2 ± 3.0
HbA1c (%)	10.5 ± 3.1
Duration of diabetes (years)	4.8 ± 4.0
Systolic blood pressure (mmHg)	121.1 ± 13.3
Diastolic blood pressure (mmHg)	77.7 ± 12.0
LDL cholesterol (mM)	2.9 ± 1.3
HDL cholesterol (mM)	1.3 ± 0.4
Triglyceride (mM)	1.8 ± 1.1
eGFR (mL/min/1.73 m^2^)	99.4 ± 21.1

Values are presented as mean ± standard deviation (SD)

*FPG* fasting plasma glucose

**Table 2:** Parameters of haematologic data, CGMs and T-TAS before and after glycemic control in STUDY 1

	Baseline	After control	*P*-value
FPG (mM)	9.6 ± 2.3	6.3 ± 1.4	0.004
Hematologic parameters			
WBC count (/μL)	5752 ± 1440	5468 ± 1342	0.191
Ht (%)	41.1 ± 4.8	40.7 ± 4.9	0.523
Plt count (10^4^ /μL)	19.3 (16.6–24.2)	20.2 (18.9–24.1)	0.683
PAI-1 (ng/mL)	24.0 (16.3–45.3)	21.0 (14.5–28.5)	0.211
Fibrinogen (g/dL)	3.3 ± 0.7	3.6 ± 0.3	0.128
Factor VII (%)	85.4 ± 8.6	83.6 ± 11.2	0.522
Factor VIII (%)	101.1 ± 33.4	92.3 ± 28.7	0.288
F1 + 2 (pM)	135.5 (105.0–169.3)	139.5 (107.3–173.5)	0.580
CGMs			
Mean PG (mM)	10.1 ± 2.5	6.8 ± 0.9	0.005
Peak PG (mM)	16.3 (11.3–19.1)	10.2 (8.8–12.4)	0.017
SD (mM)	1.8 (1.2–2.4)	1.1 (0.9–1.7)	0.242
MAGE (mM)	4.3 (2.9–7.0)	2.8 (2.2–4.1)	0.310
T-TAS			
PL-T10 (s)	125.5 (110.8–141.0)	126.0 (120.5–135.3)	0.881
PL-OT (s)	327.8 ± 67.6	308.7 ± 56.5	0.166
PL-AUC10 (kPa × min)	393.2 ± 45.3	398.3 ± 32.2	0.647

Values are presented as mean ± standard deviation (SD) or median (quartile range)

*WBC* white blood cell, *Ht* hematocrit, *Plt* platelet, *PAI-1* plasminogen activator inhibitor-1, *F1 + 2* prothrombin fragment 1 + 2, *FPG* fasting plasma glucose, *MAGE* mean amplitude of glycemic excursions, *T10* time to 10 kPa, *OT* occlusion time, *AUC10* area under the curve in 10 min

Hematologically, no significant changes were observed in WBC count, Ht level, platelet count, fibrinolysis marker (PAI-1) level and coagulation marker (fibrinogen, FVII, FVIII, F1 + 2) levels (Table 2).

In STUDY 1, the T-TAS and two microchips were used to evaluate thrombus formation. Inside the PL chip, activation of the platelet is triggered on the surface of collagen [13]. Inside the other AR chip, activation of both platelets and coagulation is triggered by collagen and tissue thromboplastin [11]. We evaluated thrombogenicity by assessing the changes in flow pressure during thrombus formation.

**Fig. 1:**
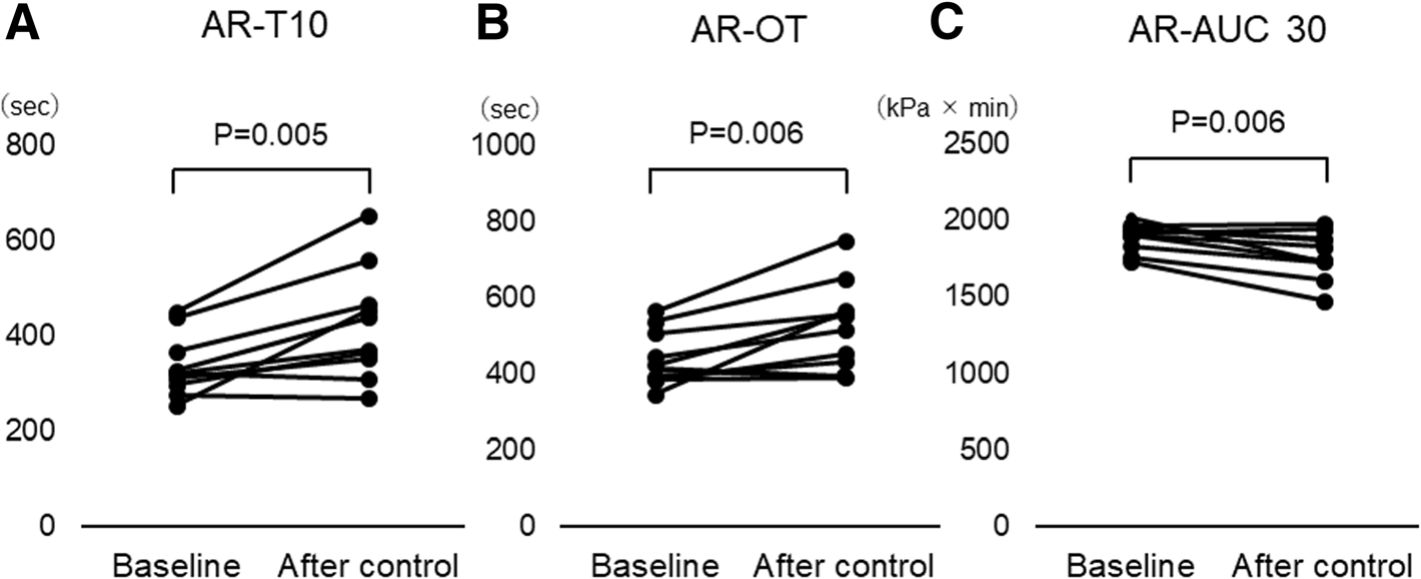
Changes in time for the flow pressure to reach 10 kPa (AR-T10) (**a**), time to occlusion (AR-OT) (**b**) and area under the flow curve at 30 min (AR-AUC 30) (**c**) before and after glycemic control in STUDY 1. Data (*n* = 10) were evaluated by two-tailed t-test

AR-T10 and AR-OT increased from 337.8 ± 64.7 s to 425.2 ± 116.9 s (*P* = 0.005) and from 441.2 ± 73.0 s to 527.7 ± 115.4 s (*P* = 0.006), respectively, while AR-AUC30 reduced from 1890.3 ± 91.3 to 1775.9 ± 154.5 kPa × min (P = 0.006) (Fig. 1). No PL chip parameters changed (Table 2).

**Fig. 2:**
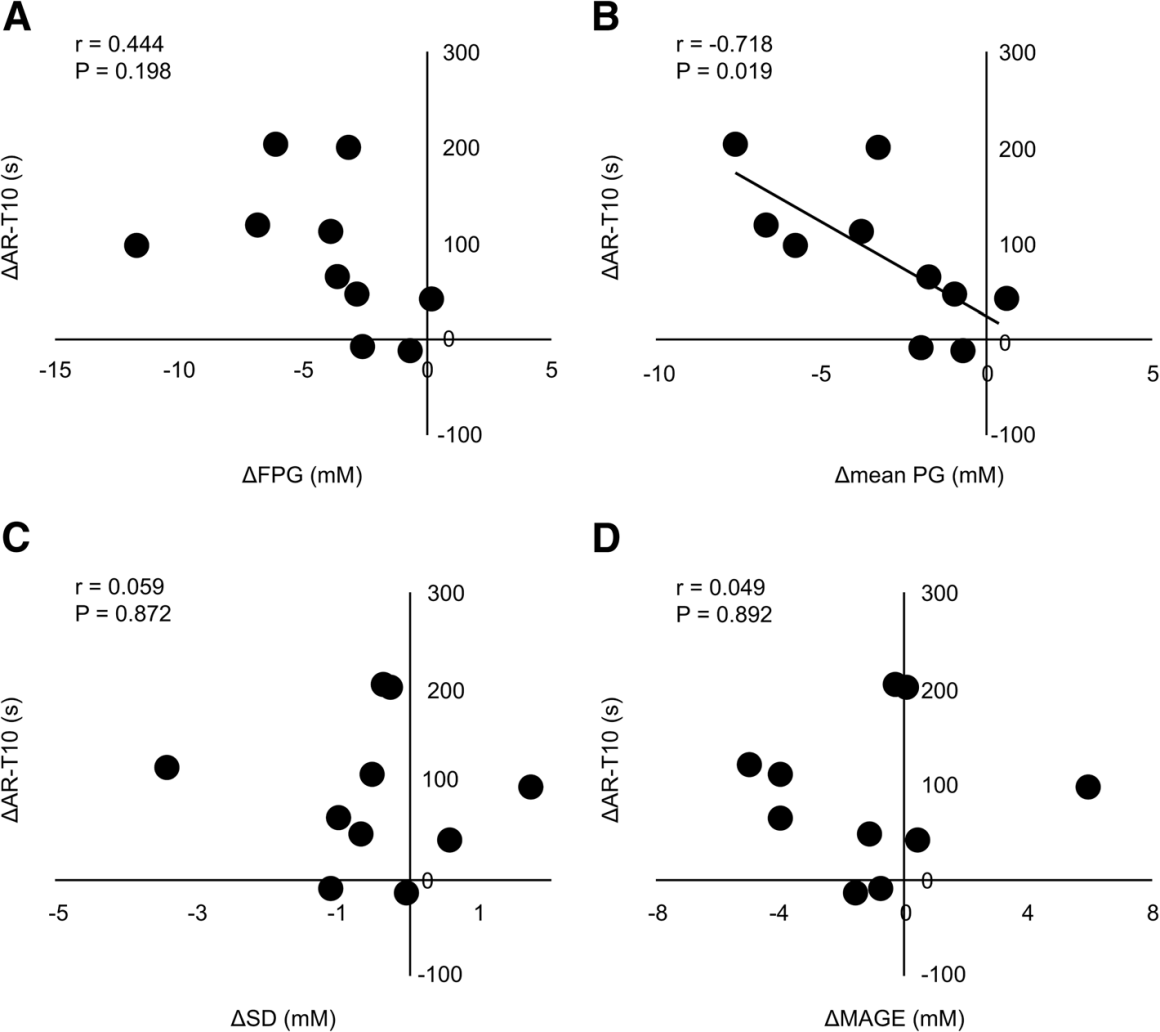
Correlation between the change in AR-T10 and the change in FPG (**a**), the change in mean BG (**b**), the change in SD (**c**), or the change in MAGE (**d**) in STUDY 1. Pearson’s rank correlation was used for assessing correlation (*n* = 10)

We compared the change in the mean PG (Δmean PG) and SD (ΔSD) with the change in T-TAS parameters (Fig. 2). The change in AR-T10 (ΔAR-T10) was significantly correlated with Δmean PG after treatment (r = − 0.718, *P* < 0.05), but not with FPG, SD and MAGE.

Although the correlation between Δmean PG and ΔAR-OT or ΔAR-AUC30 was not statistically significant, non-significant trend was observed. Additional file 1: Table S2 presents individual data on mean PG, AR-T10, AR-OT and AR-AUC30 in STUDY 1.

### Study 2

We included 10 patients who underwent an insulin tolerance test for hypothalamic pituitary evaluation (Table 3). After insulin injection, PG decreased from 5.2 ± 0.7 mM (baseline) to 1.7 ± 0.5 mM (45 min), and Ht increased from 37.5 ± 3.5% to 39.1 ± 3.7% (*P* < 0.01) and epinephrine increased from 19.5 ± 11.2 pg/mL to 449.5 ± 289.6 pg/mL (P < 0.01). However, the platelet count did not change (24.5 × 10^4^/μL to 25.8 × 10^4^/μL, *P* = 0.053).

**Table 3:** Patient characteristics in STUDY 2

Age (years)	46.8 ± 14.8
Sex (male/female)	3 / 7
BMI (kg/m^2^)	24.1 ± 3.3
HbA1c (%)	5.7 ± 0.5
Systolic blood pressure (mmHg)	121.7 ± 14.5
Diastolic blood pressure (mmHg)	75.6 ± 11.5
eGFR (mL/min/1.73 m^2^)	97.3 ± 18.6

Values are presented as mean ± standard deviation (SD)

*FPG* fasting plasma glucose; BMI, body mass index

**Fig. 3:**
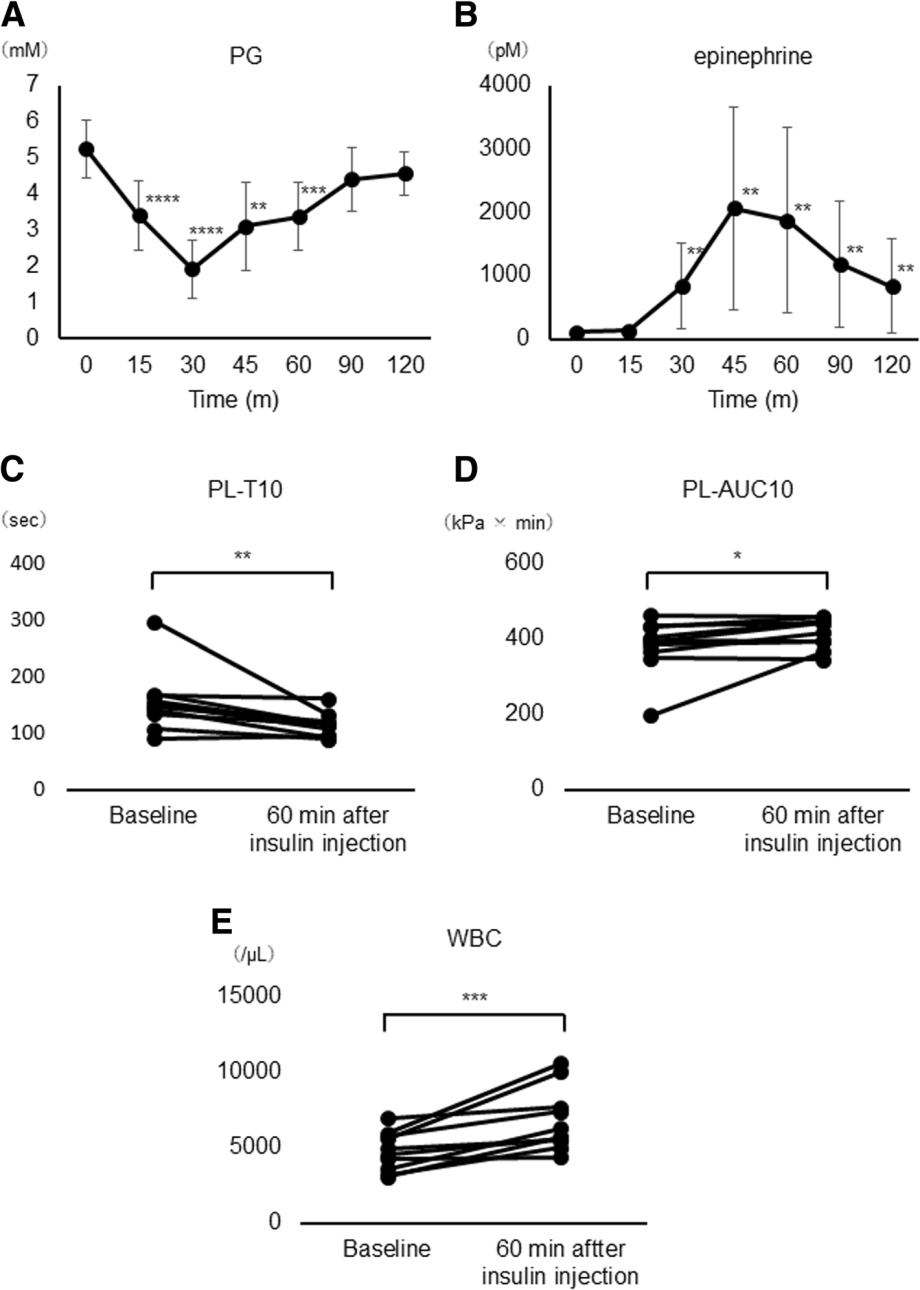
Time course of PG (**a**) and epinephrine (**b**) and changes in PL-T10 (**c**), PL-AUC 10 (**d**), WBC (**e**) before and 60 min after insulin injection in insulin tolerance test (STUDY 2). Data are presented as mean ± SD. *P < 0.05 vs basal, **P < 0.01 vs basal, ***P < 0.005 vs basal, **** *P* < 0.001 vs basal. The change in WBC was evaluated by two-tailed t-test, the others were evaluated by Wilcoxon signed rank test (*n* = 10)

PL-T10 decreased from 156.4 ± 55.5 s to 109.7 ± 15.2 s (P < 0.05), while PL-AUC10 increased from 380.3 ± 72.4 s to 428.3 ± 31.9 s (P < 0.05). PL-T10 was significantly lower, and PL-AUC10 was significantly higher, at 60 min compared to the baseline values (Fig. 3c, d).

**Fig. 4:**
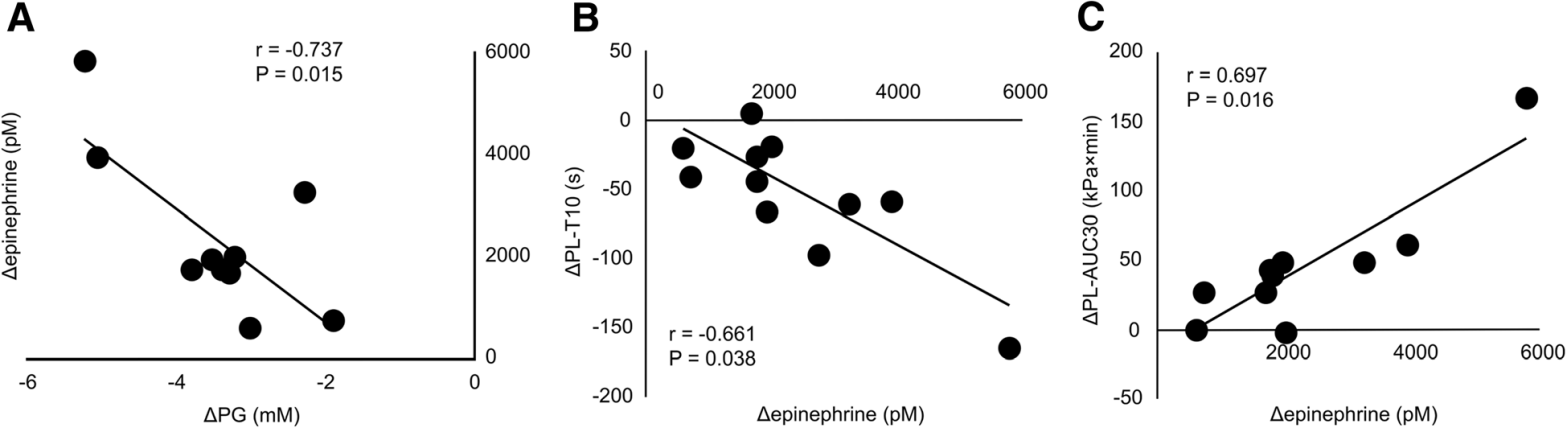
Correlation between the change in PG and the change in epinephrine (**a**), the change in epinephrine and the change in PL-T10 (**b**) and the change in epinephrine and the change in PL-AUC10 (c) in STUDY 2. Each data is the difference between the value before administration of insulin and the bottom value (PG and PL-T10) or the peak value (epinephrine and PL-AUC10) at any time point after administration of insulin. Correlation between the change in PG and the change in epinephrine was analzsed by Pearson’s rank correlation, and others were analyzed by Spearman’s rank correlation (*n* = 10)

Along with hypoglycemia, epinephrine and WBC count significantly changed (Fig. 3e). In addition, there was a correlation between the decrease in PG (ΔPG) and the change in epinephrine (Δepinephrine). Furthermore, Δepinephrine was correlated with ΔPL-T10 and ΔPL-AUC10 (Fig. 4). We found the significant correlations between ΔPL-T10 and change in Ht (r = − 0.772, *P* = 0.009), change in WBC count (*r* = − 0.915, *P* < 0.001) or change in platelet count (r = − 0.648, *P* = 0.043) (Additional file 1: Figure S2). Additional file 1: Table S3 shows individual data on the PG, epinephrine, PL-T10 and PL-AUC10 in STUDY2.

## Discussion

We aimed to elucidate the effects of short-term glycemic control on blood thrombogenicity in patients with diabetes (STUDY1) and change of platelet function during hypoglycemia (STUDY2) using a newly-developed, microchip-based flow chamber system. We used two types of microchips [13]: the PL chip to evaluate thrombogenicity, mainly involving platelets, and the AR chip to evaluate platelet activation and the coagulo-fibrinolytic reaction [11].

Although it has been reported that thrombogenic potential improves as glycemic control improves, for at least several months [15], it was not clear whether short-term control reduces thrombus formation. Our study (STUDY1) demonstrated that short-term glycemic control attenuates thrombogenicity. Of note, improvements in mean blood glucose level, but not glucose excursion, were strongly correlated with reduced thrombogenicity. In impaired glucose tolerance patients, it has been reported that suppression of postprandial hyperglycemia can prevent the onset of cardiovascular events [16]. But in type 2 diabetic patients, the protective effect of the attenuation of glucose spikes remains controversial [17]. Furthermore, there is a large-scale clinical research showing that the cardiovascular risk is lower in patients with low HbA1c values, which represent low mean blood glucose levels [18]. These results underscore the clinical significance of short-term pre-operative glycemic control to improve patients’ prognosis.

The mean value of AR-OT before glycemic control was 425.2 s, shorter than the mean value in healthy adults (489 +/− 96 s, unpublished data). After glycemic control, AR-OT improved to 527.7 s. The other AR chip parameters (AR-T10 and AR-AUC30) also improved after glycemic control. The PL chip parameters, which represent platelet-mediated thrombogenicity, showed no attenuation.

One possible explanation for the reduced thrombogenicity of the AR chip, without attenuation of PL chip thrombogenicity and coagulo-fibrinolysis markers, is glycation of fibrinogen and plasminogen because of high blood glucose. In the studies that used the purified-fibrinogen model, chronic hyperglycemia accelerated glycation of fibrinogen non-enzymatically. Glycated fibrinogen rapidly constructed a fibrin network, increasing the resistance to the fibrinolytic system, compared to non-diabetic patients [19, 20], due to changes in the kinetics of fibrin polymerization [20]. Past reports found that patients with diabetes and rapidly-improving glycemic control had significantly decreased glycated fibrinogen over 3 days [21]. In patients with diabetes, glycation of plasminogen reduced plasmin generation and impaired plasminogen function, both of which were ameliorated by glycemic control [22]. Based on these results, the hypo-fibrinolytic state may have improved secondary to decreases in glycated fibrinogen and plasminogen, even during short-term glycemic control.

It is not clear what factor, among all those altered by comprehensive diabetes care, contributed to the attenuation of thrombotic occlusion. Since the decrease in the mean PG change showed a relatively strong negative correlation with the change in AR-T10, mean PG might be an important factor. However, it is also possible that some specific treatment options, such as metformin, glucagon-like peptide-1 receptor agonists or statins, contribute to reduction of thrombogenic potentials. A large-scale study with a stratified analysis is necessary to examine this issue further.

The findings of this study differ from the findings of previous studies [23, 24] of the relationship between glycemic control and thrombogenicity. Several reported that levels of fibrinogen, PAI-1, FVII, FVIII and F1 + 2 are increased in patients with diabetes [25]; however, in the present study, the levels were within normal limits and did not change after glycemic control. These markers are influenced by various factors, such as low-grade inflammation [26], insulin resistance [27] and hyperglycemia itself [28]; therefore, our results may reflect differences in patients’ backgrounds.

The relationship between glycemic control and platelet function is controversial [29, 30]. Factors other than the glucose level may be involved in platelet function among patients with type 2 diabetes [31]. In a previous clinical study, aspirin administered for primary prevention in patients with type 2 diabetes failed to reduce the risk of macrovascular events [32]. The role of platelets in thrombus formation, among patients with type 2 diabetes, may be limited.

Conventionally, there is a recognized relationship between hypoglycaemia and increased platelet function [33]. However, prior results were verified in the absence of blood flow or with supra-physiological concentrations. Moreover, the samples consisted of platelet-rich plasma (PRP) and not whole blood. To the best of our knowledge, this is the first study to evaluate thrombus formation associated with hypoglycaemia using a flow chamber system with microchips simulated pathological blood vessels.

Platelet function was enhanced with decreasing blood glucose, and increased platelet function was strongly correlated with an increase in epinephrine (STUDY2). Several studies have shown that plasma epinephrine may have an important role in platelet activation during hypoglycemia in patients with type 2 diabetes [34, 35]. Epinephrine is secreted from the adrenal medulla through the activation of the sympathetic nervous system as a physiological defence when PG levels fall [36]. Increased epinephrine levels cause platelet aggregation through the activation of α2-adrenergic receptors [37]**.** Additionally, the changes in WBC count and Ht levels correlated with changes in PL chip parameters. Increased WBC count, partially that induced by epinephrine, may contribute to thrombus formation and progress. WBCs, especially monocytes and neutrophils, induce thrombus formation in collaboration with platelets [38]. Furthermore, increased Ht level after insulin injection could have affected blood viscosity inside the PL chip.

Our patients included one with acromegaly and one with adrenal insufficiency; however, there was no association of cortisol and growth hormone levels with platelet function [39].

It has been reported that, in patients with acute coronary syndrome, hyperglycemia is associated with poor prognosis and high rates of subsequent ischaemic events [40]. However, there is no firm evidence that glycemic control improves prognosis in patients who have experienced cardiovascular events. Our results suggest that glycemic control improves prognosis in critical situations and underscore the importance of avoiding hypoglycemia.

Although there is no study on the relationship between glycemic control and development of VTE, glycemic control-related improvements in the hypo-fibrinolysis state may lower the risk of VTE while improving prognosis.

Earlier studies investigated the effects of anti-platelet or anti-coagulant drugs on T-TAS measurements [41, 42]. One study that evaluated the effects of warfarin or direct-acting oral coagulants (DOACs) with T-TAS [43] showed a decrease in AR-AUC30, similar to the results of STUDY 1. Changes in T-TAS parameters, obtained by comprehensive diabetes care involving short-term glycemic control, appear physiologically-relevant.

Our study has several limitations. First, the number of participants was small. Nevertheless, we think that our preliminary findings are worth reporting and may inform future, more scientifically-rigorous studies. Second, we enrolled patients without diabetes in STUDY 2, because patients with diabetes have increased risk of ischaemic events associated with hypoglycaemia. Third, we did not evaluate thrombus formation with the AR chip in STUDY2. Interpretation of AR chip results might be difficult if significant changes in platelet function are observed because the AR chip results reflected either platelet aggregation or coagulation factor. Finally, we could not obtain direct evidence of glycated state alteration.

## Conclusions

In summary, we demonstrated attenuation in thrombogenicity with comprehensive diabetes care and enhancement in thrombogenicity with hypoglycemia, using a new flow chamber system. To confirm these findings, further large-scale clinical studies are needed.

## Additional files

Additional file 1: **Table S1.** Glucose-lowering medication and lipid-lowering drugs before and after glycemic control in STUDY1. **Table S2.** Changes in the mean PG, AR-T10, AR-OT and AR-AUC30 in STUDY1. **Table S3.** Changes in the PG, epinephrine, PL-T10 and PL-AUC10 in STUDY2. **Figure S1.** Intra-assay precision of the T-TAS AR assay (A) and the PL assay (B) was analyzed using a whole blood sample. **Figure S2.** Correlations between the change in PL-T10 and the change in Ht (A), WBC count (B), or platelet count (C) in STUDY2. Spearman's rank correlation was used for assessing correlation (*n*=10) (DOCX 254 kb)

## Data Availability

All data in this study are included in this article or are available from the corresponding author upon reasonable request.

## Abbreviations

AR-AUC30: area under the flow curve in 30 min
AR-OT: occlusion time: time to 80 kPa
AR-T10: time to 10 kPa
CGM: Continuous glucose monitoring
DOAC: Direct-acting oral coagulants
F1 + 2: Prothrombin fragment 1 + 2
FPG: Fasting plasma glucose
FVII: Factor VII
FVIII: Factor VIII
Ht: Hematocrit
MAGE: Mean amplitude of glycemic excursion
PAI-1: Plasminogen activator 1
PL-AUC10: area under the flow curve in 10 min
PL-OT: occlusion time: time to 60 kPa
PL-T10: time to 10 kPa
PRP: Platelet-rich plasma
SD: Standard deviation
T-TAS: Total Thrombus formation Analysis System
WBC: White blood cell
