# Supplementary material for: Effects of glycemic control and hypoglycemia on Thrombus formation assessed using automated microchip flow chamber system: an exploratory observational study
Source: Thromb J. 2019 Sep 2;17:17. doi: 10.1186/s12959-019-0206-8 (PMC6717975; doi:10.1186/s12959-019-0206-8)
Supplement: Supplementary file 1 — Table S1. Glucose-lowering medication and lipid-lowering drugs before and after glycemic control in STUDY1. Table S2. Changes in the mean PG, AR-T10, AR-OT and AR-AUC30 in STUDY1. Table S3. Changes in the PG, epinephrine, PL-T10 and PL-AUC10 in STUDY2. Figure S1. Intra-assay precision of the T-TAS AR assay (A) and the PL assay (B) was analyzed using a whole blood sample. Figure S2. Correlations between the change in PL-T10 and the change in Ht (A), WBC count (B), or platelet count (C) in STUDY2. Spearman's rank correlation was used for assessing correlation (n=10) (DOCX 254 kb) [file 12959_2019_206_MOESM1_ESM.docx]

**Additional file 1: Table S1. Glucose-lowering medication and lipid-lowering drugs before and after glycemic control in STUDY 1**.

|  |  | Baseline | After control |
| --- | --- | --- | --- |
| Case 1 | Glucose-lowering medication | Sitagliptin 50 mg | Insulin (TDD 16 U) |
|  | Lipid-lowering drugs | None | None |
| Case 2 | Glucose-lowering medication | None | Metformin 500 mg  Voglibose 0.6 mg |
|  | Lipid-lowering drugs | None | None |
| Case 3 | Glucose-lowering medication | None | Insulin (TDD 27 U) |
|  | Lipid-lowering drugs | None | None |
| Case 4 | Glucose-lowering medication | Insulin (TDD 53 U) | Insulin (TDD 52 U) |
|  | Lipid-lowering drugs | Rosuvastatin 2.5 mg | Rosuvastatin 2.5 mg |
| Case 5 | Glucose-lowering medication | Insulin (TDD 32 U)  Vildagliptin 100 mg | Insulin (TDD 46 U)  Vildagliptin 100 mg |
|  | Lipid-lowering drugs | Atorvastatin 5 mg | Atorvastatin 5 mg |
| Case 6 | Glucose-lowering medication | Insulin (TDD 20 U)  Glimepiride　1 mg  Pioglitazone 15 mg  Metformin 750 mg  Voglibose 0.9 mg | Liraglutide 0.6 mg  Glimepiride　0.5 mg |
|  | Lipid-lowering drugs | Atorvastatin 10 mg | Atorvastatin 10 mg |
| Case 7 | Glucose-lowering medication | None | Insulin (TDD 9 U)  Teneligliptin 20 mg |
|  | Lipid-lowering drugs | None | Rosuvastatin 2.5 mg |
| Case 8 | Glucose-lowering medication | Insulin (TDD 6 U)  Metformin 500 mg  Voglibose 0.6 mg | Liraglutide 0.6 mg  Metformin 1000 mg |
|  | Lipid-lowering drugs | None | None |
| Case 9 | Glucose-lowering medication | Insulin (TDD 17 U) | Liraglutide 0.3 mg  Metformin 1000 mg |
|  | Lipid-lowering drugs | None | None |
| Case 10 | Glucose-lowering medication | Insulin (TDD 18 U)  Metformin 1500 mg | Liraglutide 0.3 mg  Metformin 1500 mg |
|  | Lipid-lowering drugs | None | None |

TDD, total daily dose

|  | mean PG (mM) | | | AR-T10 (s) | | | AR-OT (s) | | | AR-AUC30 (kPa × min) | | |
| --- | --- | --- | --- | --- | --- | --- | --- | --- | --- | --- | --- | --- |
|  | Baseline | After control | Δmean PG | Baseline | After control | ΔAR-T10 | Baseline | After control | ΔAR-OT | Baseline | After control | ΔAR-AUC30 |
| Case 1 | 10.3 | 8.3 | -2.0 | 277 | 270 | -7 | 389 | 391 | 2 | 1970.2 | 1978.2 | 8.0 |
| Case 2 | 7.3 | 6.6 | -0.7 | 322 | 311 | -11 | 414 | 394 | -20 | 1915.8 | 1938.2 | 22.4 |
| Case 3 | 9.5 | 7.7 | -1.8 | 299 | 365 | 66 | 394 | 434 | 40 | 1943.7 | 1878.6 | -65.1 |
| Case 4 | 12.3 | 5.7 | -6.6 | 440 | 560 | 120 | 540 | 652 | 112 | 1758.0 | 1607.4 | -150.6 |
| Case 5 | 13.3 | 7.5 | -5.8 | 369 | 467 | 98 | 508 | 557 | 49 | 1832.1 | 1727.0 | -105.1 |
| Case 6 | 6.1 | 6.7 | 0. 6 | 312 | 354 | 42 | 445 | 517 | 72 | 1900.9 | 1824.6 | -76.3 |
| Case 7 | 11.8 | 7.9 | -3.9 | 329 | 441 | 112 | 425 | 558 | 133 | 1903.6 | 1740.1 | -163.5 |
| Case 8 | 7.2 | 6.2 | -1.0 | 323 | 372 | 49 | 384 | 455 | 71 | 1939.6 | 1866.3 | -73.3 |
| Case 9 | 13.0 | 5.4 | -7.6 | 452 | 656 | 204 | 566 | 752 | 186 | 1726.3 | 1472.7 | -253.6 |
| Case 10 | 10.2 | 6.9 | -3.3 | 255 | 456 | 201 | 347 | 567 | 200 | 2011.1 | 1726.3 | -284.8 |

**Table S2. Changes in the mean PG, AR-T10, AR-OT and AR-AUC30 in STUDY1.**

**Table S3. Changes in the PG, epinephrine, PL-T10 and PL-AUC10 in STUDY2.**

|  | PG (mM) | | | | epinephrine (pM) | | | PL-T10 (s) | | | PL-AUC10 (kPa × min) | | | |
| --- | --- | --- | --- | --- | --- | --- | --- | --- | --- | --- | --- | --- | --- | --- |
|  | Baseline | Nadir | ΔPG | | Baseline | Peak | Δepinephrine | Baseline | 60 min after | ΔPL-T10 | Baseline | | 60 min after | ΔPL-AUC10 |
| Case 1 | 4.7 | 2.6 | | -1.9 | 60.1 | 791.6 | 731.5 | 156 | 115 | -41 | 429.7 | 455.9 | | 26.2 |
| Case 2 | 4.6 | 1.6 | | -3.0 | 92.8 | 687.8 | 595.0 | 135 | 115 | -20 | 396.9 | 391.5 | | -0.3 |
| Case 3 | 4.8 | 1.4 | | -3.4 | 109.2 | 1888.8 | 1779.6 | 152 | 109 | -43 | 382.6 | 392.4 | | 40.6 |
| Case 4* | 7.2 | 1.9 | | -5.2 | 152.9 | 5944.9 | 5792.0 | 298 | 133 | -165 | 197.4 | 364.9 | | 167.5 |
| Case 5 | 5.0 | 1.5 | | -3.5 | 136.5 | 2079.9 | 1943.4 | 166 | 162 | -65 | 401.3 | 444.8 | | 50.1 |
| Case 6 | 5.2 | 1.9 | | -3.3 | 245.7 | 1501.2 | 1686.8 | 91 | 96 | 5 | 432.6 | 441.3 | | 26.1 |
| Case 7 | 5.5 | 1.7 | | -3.8 | 103.7 | 1872.4 | 1768.7 | 147 | 121 | -26 | 349.1 | 343.6 | | 44.0 |
| Case 8 | 5.9 | 0.8 | | -5.1 | 109.2 | 4039.7 | 3930.5 | 142 | 92 | -58 | 391.6 | 440.7 | | 62.0 |
| Case 9 | 4.6 | 1.4 | | -3.2 | 27.3 | 2030.8 | 2003.5 | 108 | 90 | -18 | 461 | 458.8 | | -1.1 |
| Case 10 | 5.1 | 2.8 | | -2.3 | 27.3 | 3270.9 | 3242.6 | 169 | 117 | -62 | 366.1 | 414.7 | | 48.6 |

Significant changes were observed in PL-T10 (n = 10, P = 0.009, Wilcoxon signed rank test) and PL-AUC10 (n = 10, P = 0.028, Wilcoxon signed rank test) following insulin injection. Significant changes were also observed following insulin injection in PL-T10 (n = 9, P = 0.003, two-tailed paired t-test) and PL-AUC10 (n =9, P = 0.037, two-tailed paired t-test) without Case 4* which **s**howed the most pronounced change in T-TAS parameters as well as plasma glucose and epinephrine levels.


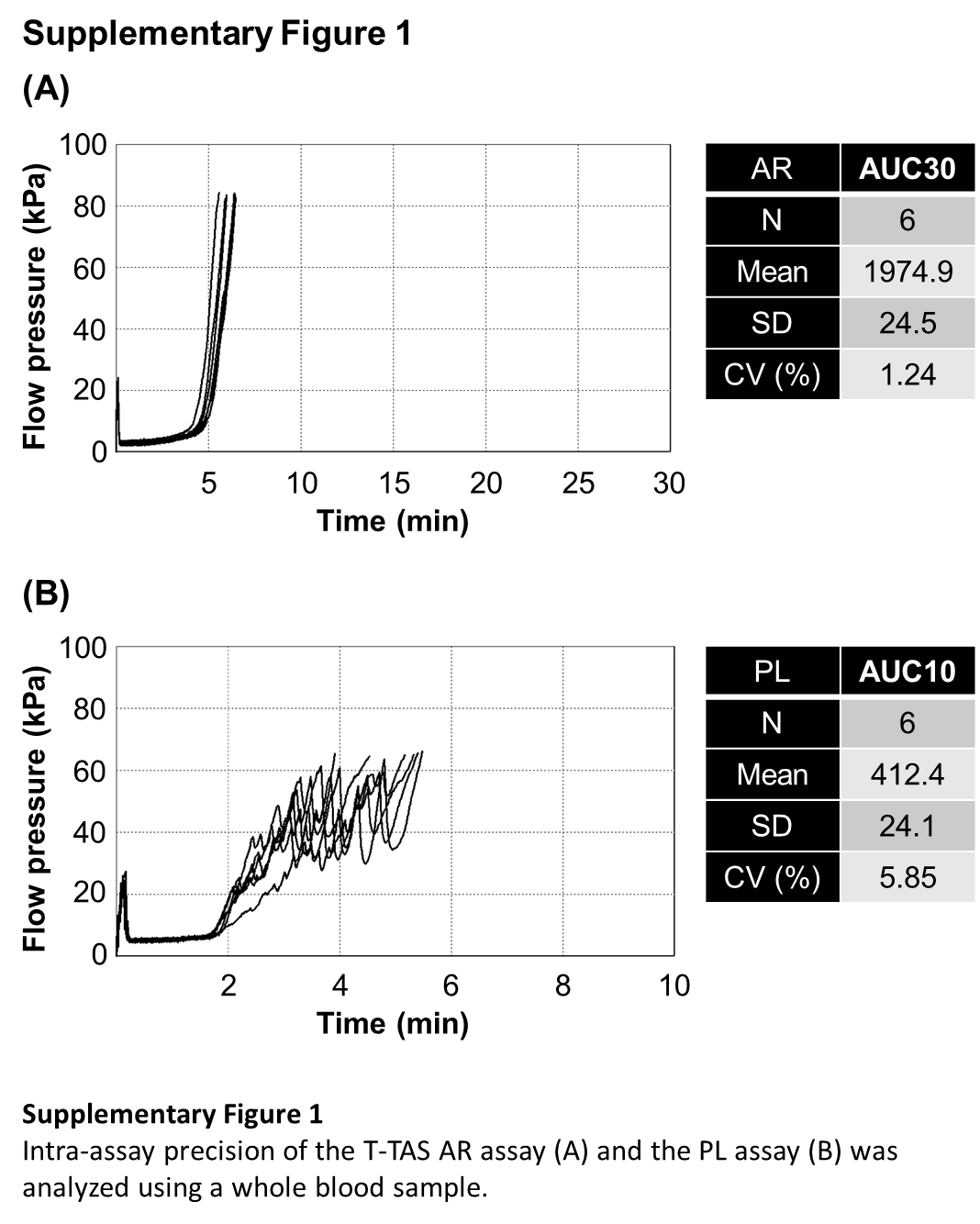


**Supplementary Figure 2**


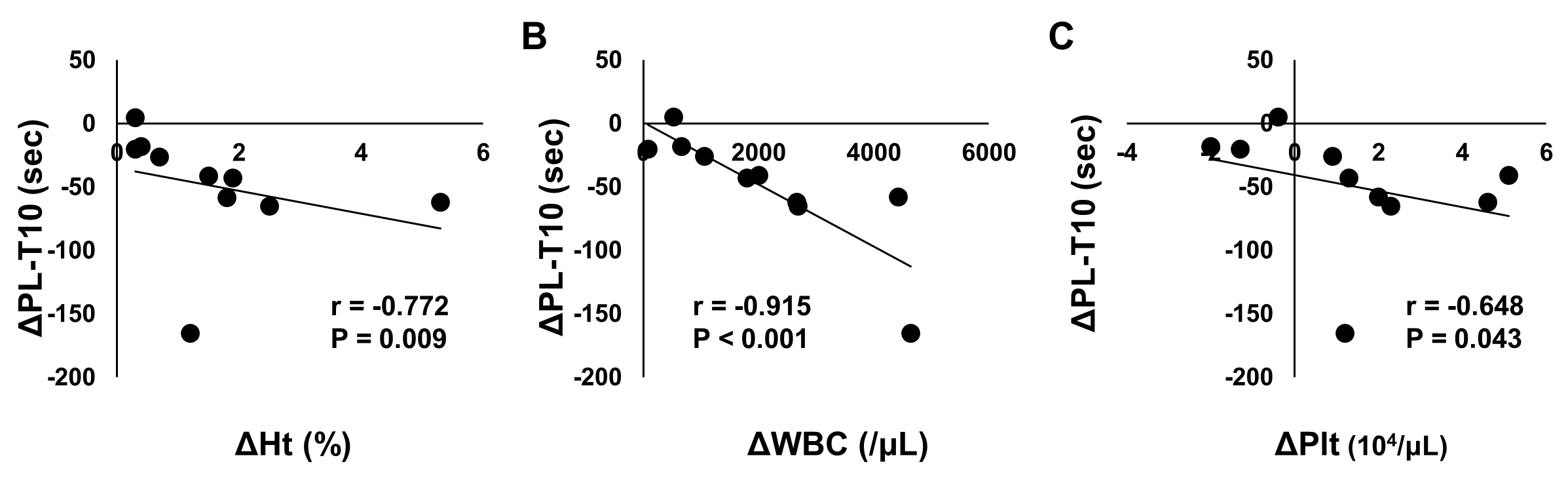


**Figure S2.**

Correlations between the change in PL-T10 and the change in Ht (A), WBC count (B), or platelet count (C) in STUDY2. Pearman’s rank correlation was used for assessing correlation (n = 10).
